# Mutation of GLP-1/Notch RAM domain results in a strong Glp-1 phenotype

**DOI:** 10.17912/micropub.biology.001391

**Published:** 2024-11-07

**Authors:** Sarah L Crittenden, Stephany J Costa Dos Santos, Sindhu Battula, Judith Kimble

**Affiliations:** 1 Department of Biochemistry, University of Wisconsin-Madison; 2 Present: WiCell Research Institute Madison, WI; 3 Present: University of Wisconsin School of Medicine

## Abstract

The distal tip cell niche uses GLP-1 /Notch signaling to maintain *C. elegans* germline stem cells. The RAM domain, which resides within the intracellular portion of the GLP-1 /Notch receptor, is integral to formation of a signaling-dependent transcription activation complex. Here we report the generation of a mutation in the GLP-1 RAM domain, created in a GLP-1 /Notch receptor with a C-terminal V5 tag. The phenotype of *glp-1 (RAM ^mut^ ) ^V5^* homozygotes is similar to that of *glp-1* null mutants, but expression of the GLP-1 (RAM ^mut^ ) ^V5^ protein was normal in the *glp-1 (RAM ^mut^ ) ^V5^* heterozygotes. We conclude that the RAM mutation abolishes receptor activity.

**Figure 1. GLP-1/Notch regulation of germline stems cells relies on the RAM domain:**
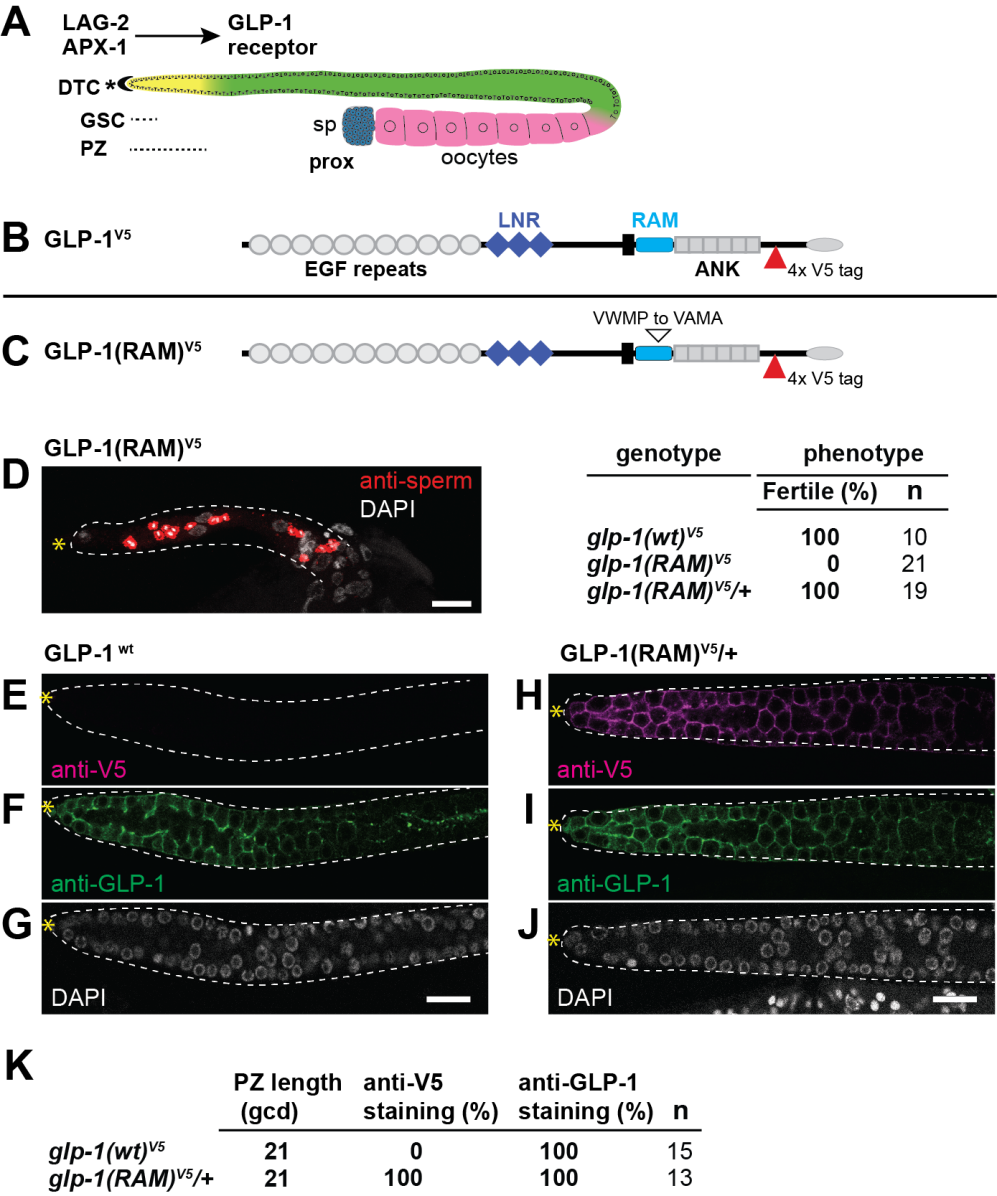
**(A)** Top, Notch ligands ( LAG-2 and APX-1 ) in the somatic distal tip cell (DTC) activate GLP-1 receptor in the germline. Bottom, hermaphrodite gonad. DTC caps the distal end of the germline and provides a niche for the germline stem cell pool (GSC; short dotted line). The progenitor zone (PZ) (long dotted line, yellow) extends from the distal end of the gonad (*) to the beginning of meiotic prophase (yellow to green). Germ cells progress through the meiotic cell cycle (green) and differentiate as sperm (blue) or oocytes (pink) in the proximal end of the gonad (prox). **(B)** Architecture of GLP-1 receptor. The extracellular domain contains EGF repeats and a set of 3 LIN-12 , Notch repeats (LNR). The intracellular domain contains a RAM domain and six ankyrin (ANK) repeats. Position of V5 tag (red triangle). **(C)** GLP-1 (RAM ^mut^ ) ^V5^ mutation. Edited amino acids are indicated (open triangle). **(D)** *glp-1 (RAM ^mut^ ) ^V5^* homozygotes are sterile. Left panel: extruded gonad containing only a few sperm (red). Right panel: Phenotype data. Fertility data from adult animals. **(E-J).** Representative images of staining in distal region of extruded gonads using anti-V5 (E, H), anti- GLP-1 (F, I) and DAPI (G, J). Gonads outlined with white dotted line, * distal end, scale bar 10 μm. **(E-G).** Gonads extruded from wildtype L4 animals. E. anti-V5 staining is undetectable (15/15 germlines). F. anti- GLP-1 detects membrane staining of wild-type untagged GLP-1 . G. DAPI-stained nuclei. **(H-J)** Gonads extruded from L4 GLP-1 (RAM ^mut^ ) *^V5^* /+ heterozygotes. H. anti-V5 detects membrane staining of GLP-1 (RAM ^mut^ ) *^V5^* protein (13/13 germlines). I. anti- GLP-1 detects membrane staining of both GLP-1 (RAM ^mut^ ) ^V5^ and wild-type untagged GLP-1 . J. DAPI-stained nuclei. **(K)** PZ data and summary of staining results obtained in extruded L4 gonads. PZ lengths were scored in germ cell diameters (gcd) from the distal end. Preliminary data from adult gonads were as follows: (1) *glp-1 (wt) ^V5^* : PZ length, 18 gcd; anti-V5 staining 0%; anti- GLP-1 staining 100%, n=10; and *glp-1 (RAM ^mut^ ) ^V5^* : PZ length, 18 gcd, anti-V5 staining 100%, anti- GLP-1 staining 100% n=5.

## Description

In the nematode *Caenorhabditis elegans ,* GLP-1 /Notch signaling maintains germline stem cells (GSCs) in the Progenitor Zone (PZ) at the distal end of the gonad (Fig 1A) (see Hubbard and Schedl 2019 for review). DSL ligands, expressed in the distal tip cell (DTC) niche, activate the GLP-1 /Notch receptor in adjacent germ cells. Ligand-induced activation causes receptor cleavage to release the receptor's intracellular domain (ICD) and allow ICD translocation to the nucleus. Within the nucleus, the RAM domain of the ICD ( Figure 1B ) facilitates formation of a signaling-dependent transcriptional activation complex (Roehl et al., 1996; Wilson and Kovall, 2006) . The nuclear ICD is thus a key marker of Notch-activated cells. Nuclear ICD is notoriously difficult to detect, but it has been visualized in nematode somatic non-gonadal tissues for LIN-12 (Deng and Greenwald 2016) , in somatic gonadal cells and early embryos for GLP-1 (Sorensen et al., 2020; Gopal et al. 2021) and in various tissues from other organisms for Notch homologs (Tveriakhina, L. et al, 2024) . However, its visualization has been challenging in nematode germline stem cells (GSC) (Gutnik et al., 2018; Sorensen et al., 2020; Ferdous et al., 2024) despite active Notch signaling in those cells (Lee et al., 2016) . For LIN-12 /Notch, RAM mutations decreased receptor activity but increased abundance of the nuclear ICD (Deng and Greenwald, 2016) . This suggested that mutating the GLP-1 /Notch RAM domain might similarly increase abundance of its nuclear ICD in GSCs.

We used CRISPR/Cas9 gene editing to mutate the GLP-1 RAM domain ( Figure 1B ) in a receptor carrying a V5 tag (Sorensen et al., 2020) . We mutated the same amino acids in the GLP-1 RAM domain ( Figure 1C ) as were mutated in the LIN-12 RAM domain (Deng and Greenwald, 2016) . Similar to LIN-12 (Deng and Greenwald, 2016) , *glp-1 (RAM ^mut^ ) ^V5^* decreased receptor activity. Indeed, *glp-1 (RAM ^mut^ ) ^V5^* mutants possessed no GSCs and only a small number of sperm ( Figure 1D ), a phenotype similar to that of *glp-1* null mutants (Austin and Kimble 1987) . Because all GSCs differentiated into sperm, we could not stain GSCs for the nuclear ICD.

The null *glp-1 (RAM ^mut^ ) ^V5^* phenotype might be explained by loss of receptor activity or a loss of the receptor itself. To distinguish between these possibilities, we compared GLP-1 expression in wild-type ( Figure 1 E -G) and heterozygous GLP-1 (RAM ^mut^ ) ^V5^ /+ L4 hermaphrodites ( Figure 1 H -J). As adults, the heterozygotes were fertile ( Figure 1D ) and both L4 and adult heterozygotes had progenitor zones similar to wild-type ( Figure 1K and legend). We used anti-V5 to visualize the GLP-1 (RAM ^mutV5^ ) protein and anti- GLP-1 antibodies to visualize both wild-type GLP-1 (Crittenden et al., 1994) and GLP-1 (RAM ^mut^ ) ^V5^ proteins. Wild-type gonads had no detectable V5 staining ( Figure 1E, K ) but had membrane staining with anti- GLP-1 ( Figure 1F, K ), as expected. Gonads from *glp-1 (RAM ^mut^ ) ^V5^* /+ heterozygotes had membrane staining with anti-V5 ( Figure 1H, K ) and anti- GLP-1 , and that staining was similar to wild-type ( Figure 1F, K ).

The GLP-1 (RAM ^mut^ ) ^V5^ mutant protein was expressed and appeared normal in membranes of heterozygotes, but no nuclear ICD was detectable in our images. Perhaps an effect of the RAM mutation would have been seen if we used the additional image processing used previously to visualize GLP-1 (wt) ^V5^ nuclear ICD (Sorensen et al 2020, Ferdous et al 2023) or if we used a rescuing transgene (Sorensen et al 2020) to allow visualization of a homozygous RAM mutant. In addition, GLP-1 (wt) ^V5^ nuclear ICD was visible without additional image processing in embryonic cells (Sorensen et al 2020, Gopal et al 2021) ; we did not examine embryonic cells for the GLP-1 (RAM ^mut^ ) ^V5^ nuclear ICD. It is intriguing that germline and embryonic nuclear ICD levels differ, suggesting that regulation of ICD levels may differ in these tissues.

We conclude that the RAM mutation abolishes receptor activity but has no noticeable effect on protein stability. Generation of *glp-1 (RAM ^mut^ ) ^V5^* adds a new reagent to the growing collection of strains that can be used to assess the distribution of active GLP-1 (Gutnik et al., 2018; Sorensen et al., 2020; Shaffer and Greenwald, 2022) and address the mechanisms of ICD regulation in different tissues.

## Methods

Strains and maintenance:

Strains were grown at 20°C on NGM plates seeded with OP50 .

CRISPR/Cas9 genome editing RAM mutation:

The RAM domain mutations were generated using a co-CRISPR genome editing strategy as described (Arribere et al., 2014; Paix et al., 2015). Wildtype animals were injected with a mix containing a gene-specific crRNA (10 μM, IDT-Alt-R), unc-58 co-CRISPR crRNA (4 μM, IDT-Alt-R), tracrRNA (13.6 μM, IDT-Alt-R), gene specific repair oligo (4 μM), unc-58 repair oligo (1.34 μM), and Cas-9 protein (24.5 μM). Guide and repair sequences are given below. F1 progeny of injected hermaphrodites were screened for mutations by PCR and then sequenced. Each allele was outcrossed against wild-type twice prior to analysis.

**Immunostaining, imaging and fertility and PZ counting:**

Immunostaining and imaging was done as described. Briefly, gonads from L4 *glp-1 (RAM ^mut^ ) ^V5^ /* + heterozygotes or adult ( *glp-1 (RAM ^mut^ ) ^V5^* homozygotes were dissected in PBS containing 0.1% Tween 20 (PBST) and 0.25 mM levamisole. Gonads were fixed in 4% paraformaldehyde in PBST for 15 minutes, then permeabilized in 0.5% Triton-X100 in PBS for 15 minutes. Gonads were then blocked in 0.5% BSA in PBST and incubated overnight at 4°C with primary antibodies. After washing, gonads were incubated with secondary antibodies in PBST for 1 hour at RT, washed, mounted in ProLong Gold and cured at least overnight before imaging. Antibody concentrations: Mouse anti-sperm (SP56, Ward et al.) 1:100, Mouse anti-V5 (1:1000, Bio-Rad), Rabbit anti- GLP-1 (1:20; Crittenden et al., 1995) Secondary antibodies (1:1000; Molecular Probes/Invitrogen): Donkey anti-Mouse Alexa 647, Donkey anti-Rabbit Alexa 488, Donkey anti-Mouse Alexa 555. DAPI (1 ng/ul) was included with secondary antibodies.

Adult hermaphrodites were scored as fertile if they contained embryos in their uterus. Progenitor zone (PZ) length was determined by marking the position of meiotic entry, then counting the number of germ cell diameters (gcd) along the edge of the germline between the distal end and the position of meiotic entry (Crittenden et al., 2023) .

## Reagents

**Table:** 

**Strains**		
Strain	Genotype	Source
N2	*Caenorhabditis elegans*	CGC
JK5933	*glp-1 ( q1000 [ glp-1 ::4xV5]) III *	CGC
JK6059	glp-1 (q1035[* q1000 ] [ glp-1 ::RAM ^VWMP to VAMA^ ])III/ hT2 [ bli-4 ( e397 ) let-?( q782 ) qIs48] (I;III)	CGC

**Table:** 

**Crispr reagents**			
Edit	crRNA	Repair	Source
*glp-1* (RAM ^mut^ ) ^V5^	5'-AAA TGG TGA ACG CAA CAG TCG TTT TAG AGC TAT GC-3'	5'-GAT TCC GAC GAC CTT TCT CGT TCG TTG ATT CCA TCG GAG CCA TGG CGA CTG TTG CGT TCA CCA TTT TTC TTT TTC TAC TTC TTT CTG GA-3'	IDT

## References

[R1] Austin J, Kimble J (1987). glp-1 is required in the germ line for regulation of the decision between mitosis and meiosis in C. elegans.. Cell.

[R2] Crittenden SL, Troemel ER, Evans TC, Kimble J (1994). GLP-1 is localized to the mitotic region of the C. elegans germ line.. Development.

[R3] Crittenden SL, Seidel HS, Kimble J (2023). Analysis of the C. elegans Germline Stem Cell Pool.. Methods Mol Biol.

[R4] Deng Y, Greenwald I (2016). Determinants in the LIN-12/Notch Intracellular Domain That Govern Its Activity and Stability During Caenorhabditis elegans Vulval Development.. G3 (Bethesda).

[R5] Ferdous AS, Lynch TR, Costa Dos Santos SJ, Kapadia DH, Crittenden SL, Kimble J (2023). LST-1 is a bifunctional regulator that feeds back on Notch-dependent transcription to regulate C. elegans germline stem cells.. Proc Natl Acad Sci U S A.

[R6] Gopal S, Amran A, Elton A, Ng L, Pocock R (2021). A somatic proteoglycan controls Notch-directed germ cell fate.. Nat Commun.

[R7] Gutnik S, Thomas Y, Guo Y, Stoecklin J, Neagu A, Pintard L, Merlet J, Ciosk R (2018). PRP-19, a conserved pre-mRNA processing factor and E3 ubiquitin ligase, inhibits the nuclear accumulation of GLP-1/Notch intracellular domain.. Biol Open.

[R8] Hubbard EJA, Schedl T (2019). Biology of the
*Caenorhabditis elegans*
Germline Stem Cell System.. Genetics.

[R9] Lee C, Sorensen EB, Lynch TR, Kimble J (2016). C. elegans GLP-1/Notch activates transcription in a probability gradient across the germline stem cell pool.. Elife.

[R10] Roehl H, Bosenberg M, Blelloch R, Kimble J (1996). Roles of the RAM and ANK domains in signaling by the C. elegans GLP-1 receptor.. EMBO J.

[R11] Shaffer JM, Greenwald I (2022). SALSA, a genetically encoded biosensor for spatiotemporal quantification of Notch signal transduction in&nbsp;vivo.. Dev Cell.

[R12] Sorensen, EB, Seidel, HS, Crittenden, SL, Ballard, JH, Kimble, J. 2020. A toolkit of tagged glp-1 alleles reveals strong glp-1 expression in the germline, embryo, and spermatheca. *microPublication biology* **2020** . 10.17912/micropub.biology.000271 PMC7326335 32626848

[R13] Tveriakhina L, Scanavachi G, Egan ED, Da Cunha Correia RB, Martin AP, Rogers JM, Yodh JS, Aster JC, Kirchhausen T, Blacklow SC (2024). Temporal dynamics and stoichiometry in human Notch signaling from Notch synaptic complex formation to nuclear entry of the Notch intracellular domain.. Dev Cell.

[R14] Wilson JJ, Kovall RA (2006). Crystal structure of the CSL-Notch-Mastermind ternary complex bound to DNA.. Cell.

